# Sex Differences Outweigh Dietary Factors in Food-Related Quality of Life in Patients with Inflammatory Bowel Disease

**DOI:** 10.3390/nu17071114

**Published:** 2025-03-23

**Authors:** Lea Pueschel, Heiner Wedemeyer, Henrike Lenzen, Miriam Wiestler

**Affiliations:** 1 Department of Gastroenterology, Hepatology, Infectious Diseases and Endocrinology, Hannover Medical School, 30625 Hannover, Germany; 2 Department of Gastroenterology, Hepatology, Interventional Endoscopy and Diabetology, Academic Teaching Hospital Braunschweig, 38126 Braunschweig, Germany; 3 PRACTIS Clinician Scientist Program, Dean’s Office for Academic Career Development, Hannover Medical School, 30625 Hannover, Germany

**Keywords:** food-related quality of life, inflammatory bowel disease, sex differences, diet diversity, diet quality

## Abstract

Background: Inflammatory bowel disease (IBD), including ulcerative colitis (UC) and Crohn’s disease (CD), consists of chronic gastrointestinal inflammation, with nutrition playing a significant role in its development. IBD patients often face dietary challenges affecting their quality of life (QoL), yet research on food-related QoL (FR-QoL) and sex-specific differences is limited. It was hypothesized that dietary patterns and choices impact food-related quality of life in IBD and that these effects vary by sex. The objective of this analysis was, therefore, to evaluate the impact of diet on food-related quality of life for men and women with IBD, respectively. Methods: A monocentric, cross-sectional study at a tertiary referral center analyzed the food-related quality of life in 117 women and 116 men with IBD, with a particular focus on dietary choices and patterns. To achieve this, multiple assessment tools, including the German version of the IBD-specific Questionnaire for Food-Related Quality of Life (FR-QoL-29-German) and a validated Food Frequency Questionnaire (FFQ) for dietary behavior, were used. Clinical indices (Harvey–Bradshaw Index (HBI); Partial Mayo Score (PMS)) and biochemical markers (C-reactive protein; fecal calprotectin) were evaluated. Results: The FR-QoL-29-German sum score differed significantly between the sexes (*p* = 0.034; g = −0.3), with men showing a higher mean score. Distinct dietary patterns showed little correlation with FR-QoL for both sexes, except for a significant inverse correlation between FR-QoL and sQ-HPF scores for men (*p* = 0.021; r = −0.214) but not for women (*p* = 0.897; r = −0.012). In a logistic regression analysis that was adjusted for confounding, the impact of IBD-specific and diet-related factors on FR-QoL was assessed, and disease entity was identified as a significant influencing factor for men but not for women. In women, older age and lower body weight were associated with higher FR-QoL. Conclusions: The findings of this study indicate that dietary choices and patterns do not exhibit uniform associations with IBD-related quality of life. In addition, sex differences have been identified as a substantial factor in IBD food-related quality of life.

## 1. Introduction

Inflammatory bowel disease (IBD), comprising ulcerative colitis (UC) and Crohn’s disease (CD), is marked by persistent gastrointestinal inflammation, with nutrition assuming a substantial role in its pathogenesis [1,2,3]. The multifaceted relationship between diet, nutrition, and IBD has been the focus of extensive research; however, the precise effect of dietary interventions and specific food components, particularly with respect to sex-related disparities, remains to be elucidated. Diet is recognized as one plausible environmental triggering factor for IBD pathogenesis and onset, with the ability to directly modulate the gut microbiome [4,5]. Therefore, various dietary strategies have been explored for IBD management, including avoidant and restrictive diets as well as the Mediterranean diet, with the latter being favored as a generally beneficial diet for IBD patients [1,3,6,7]. In the context of IBD, the application of nutritional guidelines derived from those established for the general population can lead to complications. This is due to the fact that these guidelines do not take into account the distinct dietary habits and requirements of individuals with IBD. This is further compounded by evidence suggesting significant dietary disparities between male IBD patients and the general population, as well as between female IBD patients and the general population. Research has shown that there are fewer sex-related differences in diet behavior among individuals with IBD compared to the general population [8]. Nevertheless, IBD patients frequently undergo substantial alterations in their dietary routines, propelled by trepidation concerning potential symptom exacerbation associated with food intake [9,10]. This observation underscores the intricate interplay among dietary components, the manifestation of the disease, and the psychosocial dimensions that influence IBD management. Research findings indicate a heightened propensity among IBD patients to engage in problematic eating patterns [11,12]. The multifaceted nature of the condition, with its dietary restrictions aimed at preventing or managing disease flares, can inadvertently contribute to the development of challenging eating behaviors [13], including disordered eating and dietary restrictions. An analysis revealed that 85.4% of IBD patients believed diet to be capable of triggering relapses, while 32.9% considered diet to be a potential causative agent [9]. This prevalent perspective among patients emphasizes the perceived significance of diet in IBD management and the potential psychological ramifications associated with dietary choices.

Additionally, research has indicated that up to three-quarters of IBD patients experience a decline in satisfaction with eating following their diagnosis [14]. This decline in eating satisfaction may lead to the onset of disordered eating patterns, which can, in turn, impede effective disease management. Dietary modifications, often encompassing the restriction or elimination of particular food components, may have far-reaching consequences that extend beyond basic nutritional intake and that can have a considerable impact on IBD patients’ mental health and overall quality of life [15]. Especially the implementation of restrictive diets in patients diagnosed with IBD can result in social isolation and limitations in daily activities, potentially exacerbating pre-existing mental health concerns—this is particularly concerning given the already heightened prevalence of mental health issues in the IBD population [16,17,18,19]. Research in various populations has shown that the social and psychological impact of dietary restrictions can have a significant effect on patients’ quality of life (QoL), which has been shown to be closely linked to nutritional and dietary regimens [20,21]. Consequently, the intricate relationship between IBD, diet, and psychosocial factors necessitates a comprehensive approach to patient care. Therefore, the notion of food-related quality of life (FR-QoL) has emerged as a pivotal element in the management of IBD patients [22]. A study involving 1221 IBD patients found that impaired FR-QoL is prevalent in IBD and is associated with recurrent disease flares, reduced IBD-specific QoL, and greater IBD-related distress [15]. However, research concerning the relationship between FR-QoL, dietary behaviors, and sex-related disparities in IBD remains scarce but may be crucial in optimizing patient care and improving the overall quality of life for individuals with IBD. The objective of this analysis was to provide novel insights into the impact of sex and distinct dietary patterns on the food-related quality of life of individuals diagnosed with IBD. To this end, a comprehensive evaluation of patients’ dietary habits was conducted, followed by a thorough correlation with FR-QoL with a sex-dependent focus.

## 2. Materials and Methods

The present subanalysis is part of a more extensive monocentric cross-sectional study that investigates the intersection of nutrition, psychosocial factors, and demographic characteristics of a broad IBD cohort and a healthy control population. The study’s methodology and design, including the protocol for patient screening and enrollment, are congruent with the ethical principles and standards set forth in the Declaration of Helsinki (2013). The study was conducted at a tertiary referral center, with prior approval from the Ethics Committee of Hannover Medical School (10847_BO_S_2023) and registration in the German Clinical Trials Register (DRKS) under DRKS00032771.

### 2.1. Participants and Setting

A total of 275 patients diagnosed with IBD were considered for study participation at Hannover Medical School between October 2023 and October 2024. The participants were required to provide written informed consent to be included. Study participants were further required to have a confirmed diagnosis of ulcerative colitis or Crohn’s disease for a minimum of three months, with the exclusion of individuals with conditions that precluded their participation in the study and patients under the age of 18.

### 2.2. Variables and Definition

#### 2.2.1. Data Sources/Measurements

A comprehensive data collection method was employed, entailing the administration of an online survey. The questionnaire encompassed a range of questions designed to elicit information regarding the participants’ demographic characteristics, including their sex and gender identity, age, marital status, and employment status. Data regarding height and weight were collected, and the body mass index (BMI) was subsequently determined. In addition, the handgrip strength of each participant in this study was evaluated using a hand-held dynamometer (Lafayette Instrument, Lafayette, IN, USA, Model 01165A). The risk of malnutrition was also assessed using the German version of the Malnutrition Universal Screening Tool (MUST) [23,24]. Subjects were also required to complete the German version of the Screening Questionnaire for Highly Processed Food Consumption (sQ-HPF) [25]. The sQ-HPF provides insight into participants’ consumption of processed food items. Additionally, the online questionnaire incorporated inquiries concerning the IBD-specific history, therapeutic regimens, surgical background, and comorbidities. IBD manifestation was determined using the Montreal classification for patients with Crohn’s disease and the anatomical pattern for patients with ulcerative colitis [26]. Meanwhile, disease activity and remission were determined based on the application of entity-specific disease activity index cut-offs. The Harvey–Bradshaw Index (HBI) [27] was used to assess disease activity in patients with Crohn’s disease (CD), while the Mayo score (PMS) [28] was employed for ulcerative colitis (UC).

#### 2.2.2. Food Frequency Questionnaire Variables and Macronutrients

The food frequency questionnaire (FFQ) utilized in the present study is a validated tool for dietary assessment and was initially developed for utilization in the German Health Examination Survey for Adults (DEGS), conducted from 2008 to 2011 [29]. The FFQ was administered to collect habitual dietary intake data for the previous four weeks. It was scored by calculating the mean daily quantities of specific foods and beverages [29], while nutrient intakes were determined using Federal Food Code (BLS) reference data [30]. The glycemic index was estimated by computing the FFQ variables. The estimated energy intake (EEI) and the sex-specific resting energy expenditure (REE) were expressed in kilojoules (kJ). All data pertaining to dietary, energy, and nutrient intake are estimated using the Statistical Package for the Social Sciences (SPSS) software with reference data from the validation study of the Food Frequency Questionnaire (FFQ) and Federal Food Code (BLS) reference data. The FFQ also requests that study participants disclose their dietary predilections, including whether they habitually abstained from one or more of the following foodstuffs or food categories: (1) meat, poultry, and cold cuts; (2) fish; (3) milk and dairy products; (4) eggs.

#### 2.2.3. Mediterranean Diet Score

The Mediterranean diet score (MDS) is an adherence index and was adapted from Trichopoulou et al. [31] and recalibrated based on the sex mean values for selected food groups, as reported by the FFQ. The methodology employed to calculate the Mediterranean Diet Score (MDS) for this study has been published in previous studies [8]. The total MDS score ranges from 0 to 9, with 9 representing maximum adherence to the Mediterranean diet. Subsequent categorization of the MDS has been conducted into percentiles, with a score of 3 or less signifying poor adherence and a score of 6 or more indicating high adherence.

#### 2.2.4. Diet Quality

Diet quality was computed using the German Nutrition Society (DGE) guideline for food intake [32]. If the recommended amount was observed, the diet was given 1 point; if the recommended amount was not observed, it was given 0. The diet quality score ranges from 0 to 12, with 12 points representing a high diet quality and maximal adherence to the DGE guideline. Diet quality was further coded as binary; a score of 6 or more corresponds to good diet quality.

#### 2.2.5. Diet Diversity

The German Nutrition Society (DGE) [32] guidelines were also used to calculate the dietary diversity score, with points awarded based on daily or weekly consumption of the foods or food groups listed in the guidelines. These include fruits and vegetables, juice, beans and legumes, nuts and seeds, potatoes, butter and margarine, dairy products, fish, meat and poultry, deli meats, eggs, and cereals. Consuming each of these foods counts for one point apiece. Thus, a highly diverse diet would have a score of 12 points, while a monotonous diet would have a score of 1 point. The dietary diversity score was also coded as binary, with a score of 7 and above indicating a diverse diet.

#### 2.2.6. Food-Related Quality of Life

Assessment of food-related quality of life was conducted using the German version of the FR-QoL-29 scale [33]. This patient-reported outcome measure (PROM) comprises 29 statements addressing food-related quality of life over the previous two weeks. Participants indicate their level of agreement using a five-point scale. The final score on the FR-QoL-29 ranges from 29 to 145, with 145 representing a favorable IBD food-related quality of life [22]. Participants were categorized into three percentiles based on their FR-QoL-29-German scores. The *Low* category indicates scores from 0 to 73, the *Medium* category indicates scores from 74 to 93, and the *High* category indicates scores of 94 and above. The FR-QoL-29-German score was recoded as a binary variable to facilitate logistic regression analysis, with 1 representing a score above 85 and thus indicating a high food-related quality of life as an outcome.

#### 2.2.7. Laboratory Values

As per protocol, biomaterials (blood, stool samples) were collected during the screening visit. Routine lab tests were performed, including hemoglobin levels (g/dL) and an evaluation of ferritin concentration (µg/L). Lab values further included C-reactive protein (CRP) (mg/L) and fecal calprotectin (mg/kg).

### 2.3. Statistical Analysis

Statistical analyses were performed using two software programs: the Statistical Package for the Social Sciences (SPSS), version 29.0.1.0 (SPSS, IBM, Armonk, NY, USA), and GraphPad PRISM version 10.4.0 (GraphPad Software, Boston, MA, USA). Qualitative variables are expressed in terms of total sums and proportions. The statistical significance of the baseline characteristic variables was ascertained through the implementation of either a Student’s *t*-test or Fisher’s exact test. In instances where the Bonferroni correction was deemed applicable, it was employed. In the absence of an explicit indication to the contrary, all statistical tests are two-sided. The Student’s *t*-test was employed to compare dietary variables between the sexes and within the FR-QoL-29 terciles. The calculation of Spearman’s correlation coefficient was performed to evaluate the relationship between the scores obtained from the selected dietary pattern scores and the German FR-QoL-29 score. Subsequent analysis employed multivariable logistic regression to evaluate the probability of an association between dietary and disease-related factors on food-related quality of life. The ensuing odds ratio (OR), along with the 95% confidence interval (CI) and the corresponding level of statistical significance (p), were systematically documented. A detailed exposition of the findings of the fully adjusted multivariable regression analysis can be found in the results section.

The adequacy of the fully adjusted logistic regression model, which was employed to predict outcomes pertaining to the food-related quality of life of women in relation to food consumption and disease burden, was assessed using the Omnibus Tests of Model Coefficients (*p* < 0.001), R2 (Nagelkerkes: 0.465; Cox & Snell: 0.347), and the Hosmer–Lemeshow test (*p* = 0.549). These assessments enabled the determination of the model’s goodness of fit. Consequently, the model’s performance was appraised by examining its classification table. This analysis indicated an overall accuracy of 75%. The adequacy of the fully adjusted logistic regression model was assessed using a series of statistical tests to determine its efficacy in predicting outcomes related to the food-related quality of life of men, taking into account their food consumption patterns and disease burden. This assessment utilized various metrics, including omnibus tests of model coefficients (*p* < 0.001), R2 (Nagelkerkes: 0.382; Cox & Snell: 0.285), and the Hosmer–Lemeshow test (*p* = 0.361). The model’s performance was evaluated by examining its classification table. The resultant analysis indicated an overall accuracy of 78.9%.

#### 2.3.1. Confounding Factors and Bias Risks

A thorough evaluation of the regression models was conducted to ascertain the presence of potential confounding variables. Variables included in the adjustment process spanned a range of metrics, including the disease entity, the disease activity status, age, weight, handgrip strength, the ratio of animal-to-plant-based protein intake, the estimated glycemic index per meal, the diet quality, the diet diversity, the median diet score, and the sQ-HPF score. This comprehensive approach was undertaken to ensure that the regression models were controlled for potential confounding variables. Prior to data analysis, a review was conducted to identify individuals who were actively nursing at the time of study participation, revealing two individuals who were nursing at the time of study participation. Given the markedly elevated dietary intake among nursing individuals, it was deemed necessary to exclude all such cases from further analysis to prevent any potential distortion of the results. In addition to the statistical significance (p), the estimated effect size is reported as (g) or Cramer’s V. It is imperative to acknowledge the potential for bias inherent in recall surveys. Additionally, misreporting of dietary intake in patient-reported outcomes is a prevalent issue [34]. The extent of underreporting among study participants was determined by calculating the ratio of EEI to REE, as previously outlined [35]. In order to determine the possibility of discrepancies between BMI and EEI, participants were surveyed regarding the initiation of any dietary regimens or alterations in established dietary routines within the past five weeks.

#### 2.3.2. Sample Size and Missing Data

A priori sample size estimation was based on the original study objectives, which included the investigation of sex- and disease-specific differences in nutritional and psychosocial factors in an IBD cohort and a control cohort with a distinct focus on patient profiles in association with the serum metabolome and stool microbiome. The present analysis constitutes a subanalysis of this study.

**Figure 1:**
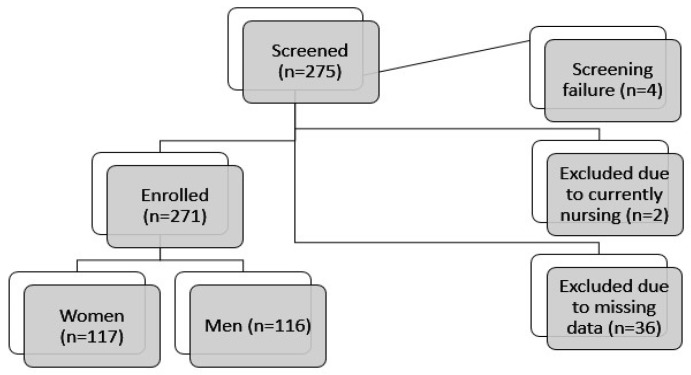
Schematic representation of the process for patient enrollment.

Of the 275 individuals diagnosed with IBD who underwent screening, four were identified as screening failures. Consequently, 271 IBD patients were enrolled in the study. Of the remaining participants, 36 were excluded from the present analysis due to the absence of requisite data, while two were excluded due to nursing (Figure 1).

## 3. Results

### 3.1. Study Population

The sex distribution was found to be well-balanced, with 50.2% of participants being women. However, the distribution of disease entities exhibited a skew, with the majority of cases being diagnosed with Crohn’s disease for women (64.1%) as well as men (56.9%). The median age for women was 38, and for men, 40. The study observed a balanced distribution of advanced drug treatments across both sexes, with a reported 57% of women and men receiving such treatments. Additionally, 52.7% of women were in remission, compared to 53.2% of men. While the majority of study participants demonstrated only a low risk for malnutrition (women: 48.7%; men: 61.2%), a comparatively higher percentage of patients were identified as being at high risk for malnutrition (women: 28.2%; men: 19.8%) as opposed to exhibiting medium risk (women: 23.1%; men: 19.0%) (Table 1).

### 3.2. Food-Related Quality of Life Score

**Figure 2:**
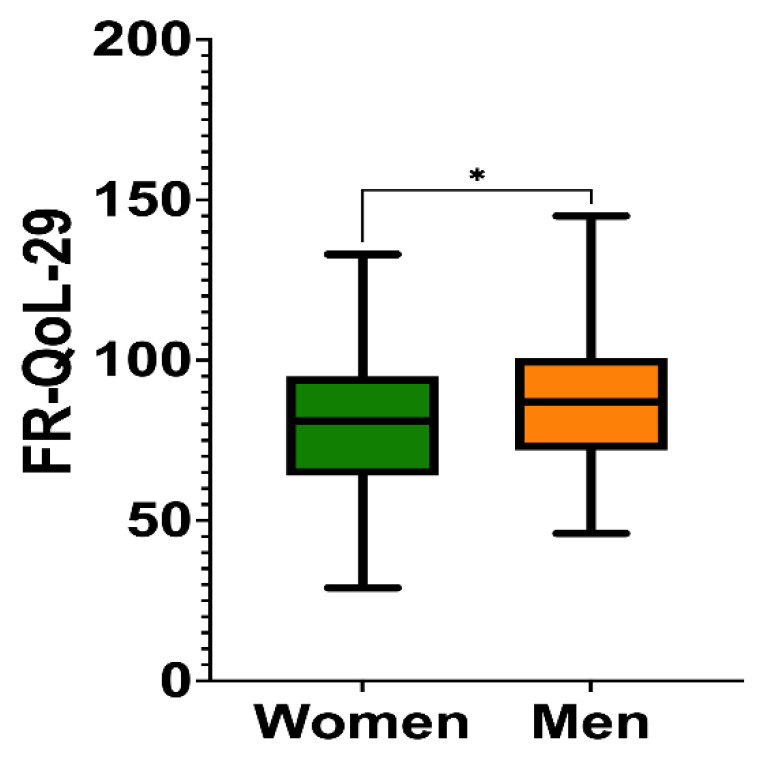
Boxplots showing food-related quality of life as measured by the FR-QoL-29 for men and women; the sum score shows statistically significant differences between the sexes (*p_t_*_-test_ = 0.034; g = −0.3). Significance level is shown with an asterisk (*) for *p* < 0.05.

In order to examine the sex differences in food-related quality of life for patients with IBD, a Student’s *t*-test was used. The FR-QoL-29-German sum score shows statistically significant differences between the sexes (*p* = 0.034; g = −0.3), with a clear trend towards a higher mean FR-QoL-29 for men (Figure 2).

### 3.3. Food-Related Quality of Life and Diet Quality

To further investigate possible sex-related differences in dietary quality and food-related quality of life, study participants were divided into three percentiles (*Low*, *Medium*, and *High*) based on the FR-QoL-29-German scoring. Using a Student *t*-test within these groups showed significant differences between the sexes that were unique for each group. In the *Low* group, the diet quality score showed that women had a higher diet quality than men (5.8 to 4.9; *p* = 0.019; g = 0.5); this was also true in the *Medium* group (5.4 to 4.5; *p* = 0.003; g = 0.7), but not in the *High* group (5.2 to 4.8; *p* = 0.226; g = 0.3). While diet diversity showed no significant differences between the sexes in the *Low* and *High* groups, respectively, it differed significantly between the sexes in the *Medium* group, with men having reported a higher diet diversity (6.1 to 5.6; *p* = 0.044; g = −0.5). Meanwhile, the Mediterranean diet score differed significantly between the sexes in the *High* group, with men having a higher mean Mediterranean diet score (4.6 to 3.6; *p* = 0.007; g = −0.6). The proportion of energy intake derived from highly processed foods, as estimated by the sQ-HPF, showed no significant differences between the sexes in all three groups (Table 2).

### 3.4. Food-Related Quality of Life and Food Groups

Possible sex-related differences in dietary choices and food-related quality of life were also investigated via Student *t*-tests. In the *Low* group, women exhibited a higher energy percentage (EN%) for fruits and vegetables than men (9.5 to 6.2; *p* = 0.036; g = 0.5), meanwhile in the *Medium* group, women had a higher daily intake of fruits and vegetables (g/d) (287.7 to 191.3; *p* = 0.043; g = 0.5). However, this did not translate to a significant difference in energy percentage (EN%) for fruits and vegetables (8.7 to 5.9; *p* = 0.054; g = 0.4). In the *Medium* group, women had a significantly higher daily intake of nuts and seeds (9 to 3.2; *p* = 0.043; g = 0.5), which was also a significantly higher energy percentage (3 to 1; *p* = 0.033; g = 0.5). In the same group, while mean daily intake did not significantly differ between the groups, men exhibited a significantly higher energy percentage of meat (13.5 to 9.4; *p* = 0.037; g = −0.5). Meanwhile, in the *High* group, men showed a significantly higher daily intake of meat (91.5 to 65.8; *p* = 0.045; g = −0.5), which was also a significantly higher energy percentage (12.2 to 8.8; *p* = 0.033; g = 0.5). In the same group, men exhibited a significantly higher energy percentage of cereal products (21.8 to 17.9; *p* = 0.045; g = −0.5) (Table 3).

### 3.5. Food-Related Quality of Life and Macronutrients

In addition, possible sex-related differences in macronutrients and food-related quality of life were also investigated via Student *t*-tests, while men in the *Low* group had a significantly higher estimated energy intake per day than women (10,531 to 7289 kJ/d; *p* = 0.009; g = −0.6); however, this was not true for the *Medium* (8391 to 7285 kJ/d; *p* = 0.158; g = −0.3) and *High* (7991 to 7437 kJ/d; *p* = 0.491; g = −0.2) groups, where men did report a higher intake but this was not significant. Meanwhile, in the *High* group, the daily intake of ethanol showed a significantly higher mean for men compared to women (60.9 to 6.5 g/d; *p* = 0.004; g = −0.6), which was also evident in the energy percentage for ethanol (23.1 to 2.7; *p* = 0.005; g = −0.6) (Table 4).

### 3.6. Correlation of Food-Related Quality of Life and Dietary Pattern Scores

**Figure 3:**
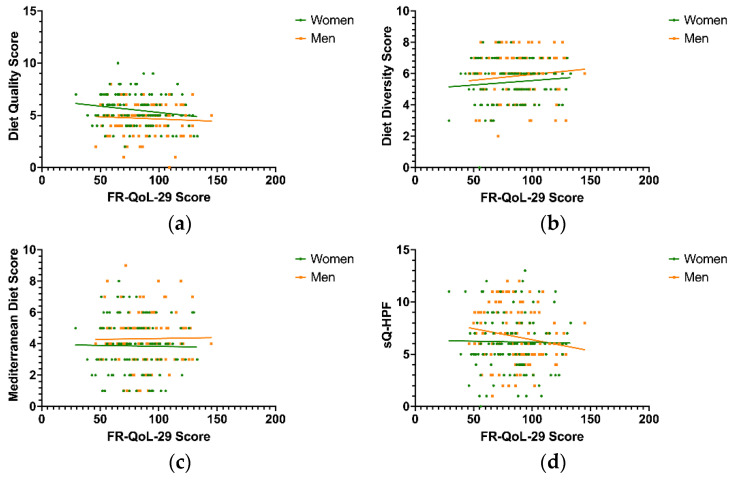
Sex-related trends and differences in correlation with food-related quality of life assessed via Spearman’s correlation coefficient: (**a**) IBD food-related quality of life did not significantly correlate with the diet quality score for men (*p* = 0.647; r = −0.043) and women (*p* = 0.052; r = −0.180); (**b**) IBD food-related quality of life did not significantly correlate with the diet diversity score for men (*p* = 0.248; r = 0.108) and women (*p* = 0.611; r = 0.048); (**c**) IBD food-related quality of life did not significantly correlate with the Mediterranean diet score for men (*p* = 0.950; r = 0.006) and women (*p* = 0.754; r = −0.029); (**d**) IBD food-related quality of life showed a significant inverse correlation with the sQ-HPF for men (*p* = 0.021; r = −0.214) but not women (*p* = 0.897; r = −0.012). sQ-HPF—Screening Questionnaire of Highly Processed Food Consumption.

To gain further insight into potential correlations of selected dietary patterns and characteristics, Spearman’s correlation coefficient was calculated for both sexes in order to assess the relationship between the scores obtained from the aforementioned selected dietary pattern scores and the FR-QoL-29-German sum score. Substantial outcomes were only attained for the sQ-HPF score of IBD men (r = −0.241) but not for women (r = −0.012). Furthermore, a positive correlation (women: r = 0.048; men: r = 0.108) was observed for diet diversity, suggesting a tendency for food-related quality of life to increase as diet diversity increases, although this trend was not statistically significant. For diet quality, however, an inverse correlation was observed (women: r = −0.180; men: r = −0.043), indicating a tendency for food-related quality of life to decrease as diet quality increases, but again this trend did not reach statistical significance. For the Mediterranean diet score, an inverse correlation was observed for women (r = −0.029) but not for men (r = 0.006); however, neither trend reached statistical significance (Figure 3a–d).

### 3.7. Influence of Diet and Disease-Specific Factors on a High Food-Related Quality of Life in Men and Women with IBD

To determine possible impact factors on food-related quality of life, an adjusted logistic regression analysis was conducted for men and women. The influence of nutritional choices and dietary patterns showed no statistically significant impact factor for men with IBD. However, disease entity (OR: 0.2; 95% CI: 0.08–0.74; *p* = 0.013) and disease status (OR: 0.1; 95% CI: 0.03–0.28; *p* < 0.001) were revealed as impacting food-related quality of life. (Table 5) The same analysis for women again showed that nutritional choices and dietary patterns did not impact food-related quality of life, but disease status (OR: 0.1; 95% CI: 0.02–0.23; *p* < 0.001) did. In addition, body weight (kg) (OR: 0.9; 95% CI: 0.91–0.99; *p* = 0.025) and age (years) (OR: 1.1; 95% CI: 1.01–1.10; *p* = 0.010) were shown to impact food-related quality of life (Table 6).

## 4. Discussion

Data on sex influences and specific dietary choices and patterns are lacking in the existing literature on food-related quality of life in inflammatory bowel disease (IBD). While low food-related quality of life has been shown to be associated with decreased intakes of specific nutrients in the IBD population [15], data on sex-related associations are lacking. Therefore, this analysis was designed to provide a comprehensive insight into the impact of sex and differing dietary choices and patterns on food-related quality of life in IBD. Although the sex distribution in our collective was well balanced, we observed significant differences in food-related quality of life between the sexes, with men having a higher general IBD-specific food-related quality of life. Although there is a lack of data from the general population on sex differences in food-related quality of life, women in different populations and settings have been shown to have lower overall quality of life (QoL) scores than men [36,37,38]. Concerning general quality of life, these findings are also in line with known data from the IBD population [39].

When examining the relationship between food-related quality of life and specific dietary aspects, a positive association was observed for dietary diversity, suggesting a tendency for food-related quality of life to increase as dietary diversity increased. However, this trend was not statistically significant. For diet quality, an inverse relationship was observed, suggesting a tendency for food-related quality of life to decrease as diet quality increases. Although this trend did not reach statistical significance, it is surprising as it is known from different populations that diet quality positively affects food-related quality of life [20,40,41,42]. In addition, specific food choice drivers have been shown to have a significant impact on food-related quality of life in a recent study of behaviors related to food-related quality of life in IBD. Patients who demonstrated a high level of health engagement had the highest food-related quality of life, suggesting a potential link between health engagement and improved well-being in patients with IBD. Furthermore, men who scored high on health engagement but low on food engagement (defined as not using food to help manage disease or mood) scored the highest.

At the same time, women who had both a high level of health involvement and a high level of food involvement were among the highest scorers [43]. This observation is consistent with previously documented data showing that men have different preferences and consumption patterns, while women, in general, have dietary habits that are more closely aligned with dietary guidelines [44,45].

Furthermore, women generally appear to follow healthier diets compared to men [44,46,47], which was also evident from the significantly higher intake of fruits and vegetables and nuts and seeds reported for women within our cohort. In addition, it is likely that men are more self-confident in their dietary desires, with a stronger adherence to dietary preferences, which may take precedence over nutritional needs [48,49]. This could be a possible explanation for the significant correlation between highly processed food and food-related quality of life for men, as well as the significant discrepancy in ethanol intake between the sexes. The analysis further showed a possible link between high FR-QoL and increased alcohol intake in men.

In contrast, a decrease in alcohol consumption and a high FR-QoL were observed in women. This sex difference was also observed for meat consumption. Men in the highest-scoring FR-QoL group reported a mean daily alcohol intake of over 60 g. This finding is of concern. Nonetheless, given the limitations of the present data, it is not possible to determine the reason for the elevated alcohol intake observed in IBD men of this cohort. Meanwhile, women in the same group reported 6.5 g of mean daily alcohol intake. This discrepancy may also provide a further explanation for the higher level of adherence to a Mediterranean diet among men, as measured by the Mediterranean Diet Score (MDS). It is noteworthy that the Mediterranean Diet Score (MDS), in addition to analogous screening tools for assessing adherence to the Mediterranean diet, considers ethanol intake within a certain range to be beneficial. In contemporary discourse, however, this perspective is no longer considered beyond reproach; rather, it necessitates further examination and deliberation.

The need for a more in-depth investigation tailored to the specific characteristics of IBD was evident, given the multifaceted aspects surrounding sex differences in food-related quality of life. In addition, while health beliefs may mediate some of the differences in dietary preferences between the sexes, they are not the sole explanation for the observed differences [44,47]. A multivariable approach showed that men and women with active disease were less likely to have a high FR-QoL compared to those in remission. For men, an entity-specific effect was observed, but not for women. In particular, men with Crohn’s disease seem to be more affected, as they were less likely to have a high food-related quality of life compared to men with ulcerative colitis. Given the well-documented disparities in the prevalence, symptoms, and progression of IBD according to sex, it is evident that men are predisposed to a heightened risk of more complicating CD disease phenotypes compared to that of females, including early-stage disease onset, upper GI involvement, penetrating behavior, and perianal disease [50], which could influence food-related quality of life, especially in CD men. No other independent associations were seen in men with IBD.

Notably, this suggests that the possible influence of psychosocial factors, such as increasing age and decreasing body weight, was independently associated with higher FR-QoL in women. In light of the documented sex-related disparities concerning IBD and its psychosocial implications, the observed discrepancy in independent associations regarding food-related quality of life among the sexes is not unexpected [39]. Evidence shows that as women age, they experience a change in perspective, focusing more on the functionality of the body and less on appearance and becoming more grateful for their health and physical abilities [51]. Higher FR-QoL in older women is also consistent with findings from an Australian longitudinal study of adults aged 55–65, where higher health-related quality of life (QoL) was associated with higher dietary quality and women’s emotional well-being [41].

Consistent with our study findings, showing that reduced body weight independently of IBD is associated with improved food-related quality of life in women with IBD, some research has confirmed the existence of an inverse relationship between increased body mass and health-related QoL [52,53], with the findings also suggesting sex-specific differences. One study reported that men with a higher BMI had a higher QoL [54], while another study showed a lower QoL for obese women but not for obese men [55]. This is consistent with known data, including a comprehensive global review of the underlying mechanisms of sex differences in dietary choices, which found that women not only have a stronger belief in the benefits of a healthy diet but also show greater commitment to body weight management [46].

In addition, it has been shown that for IBD women, their quality of life is significantly impacted by their body image [39,56]. Despite the comprehensive character of this subanalysis accounting for diverse dietary patterns and sex-related differences, this study is encumbered by certain limitations. Given the complex heterogeneity of IBD, psychosocial effects, diet, and sex, the overall sample size of this analysis is relatively small. This limitation precludes the ability to account for inherited recruitment bias, as this is a monocentric setting of a tertiary referral center. The inability to adjust for socioeconomic factors due to data availability constitutes a notable limitation since it has been established that socioeconomic factors, particularly food security, exert a significant influence on quality of life in diverse populations [57,58,59]. In addition, physical activity [60] and food beliefs [61,62] are possible contributors to the influence of food-related quality of life. Finally, given that this is a cross-sectional study, the question of whether intra-individual sex-specific trajectories in dietary patterns are associated with FR-QoL and disease activity status changes is not answerable in this setting. Consequently, future research in this field should prioritize a multicentric longitudinal design.

## 5. Conclusions

This analysis contributes to the expanding body of research on nutrition and quality of life in individuals with IBD, with a particular emphasis on the underrepresented topic of sex differences. The findings indicate that dietary choices and patterns do not exhibit uniform associations with IBD-related quality of life in men and women. In addition, the present study underscores the necessity of incorporating sex differences as a pivotal factor in the evaluation of IBD food-related quality of life. In summary, sex differences in food-related quality of life (FR-QoL) may be due to a variety of factors, including societal expectations and gender roles with respect to food consumption, as well as differences in food preferences, eating behaviors, and beliefs about health and nutrition. Furthermore, differences in women’s and men’s self-perception and responses to psychosocial stressors may contribute to these discrepancies. Overall, there is a need for a more careful and individualized approach to the care of patients with IBD, including a consideration of the patient’s sex. Our results demonstrated that dietary choices and patterns, including dietary quality and diversity, were not associated with IBD-related quality of life per se, in contrast to other populations. In addition, sex-related differences were found to be a contributing factor in IBD food-related quality of life.

## Figures and Tables

**Table 1:** Demographic data.

		Women	Men	
		(*n* = 117)	(*n* = 116)	*p*
Crohn’s disease [*n*(%)]		75 (64.1%)	66 (56.9%)	0.285
Current advanced drug therapy [*n* (%)]		65 (57%)	65 (57%)	0.999
Disease Activity [*n* (%)]	Remission	58 (52.7%)	59 (53.2%)	0.999
Location of Crohn’s [*n* (%)]	L1: ileal	17 (22.7%)	18 (27.3%)	0.999
	L2: colonic	18 (24%)	7 (10.6%)	0.302
	L3: ileocolonic	32 (42.7%)	35 (53%)	0.999
	L4: isolated upper disease	8 (10.7%)	6 (9.1%)	0.999
Crohn’s behavior [*n* (%)]	B1: nonstricturing, nonpenetrating	31 (41.3%)	20 (30.3%)	0.999
	B2: stricturing	34 (45.3%)	32 (48.5%)	0.999
	B3: penetrating	10 (13.3%)	14 (21.2%)	0.999
Ulcerative colitis: Montreal classification [*n* (%)]	Proctitis	3 (7.1%)	3 (6%)	0.999
	left-sided colitis	14 (33.3%)	18 (36%)	0.999
	pancolitis	25 (59.5%)	29 (58%)	0.999
Disease duration [median (IQR)] (years)		12 [7–20]	13 [7–19]	0.679
Surgery [*n* (%)]		39 (33.3%)	46 (39.7%)	0.343
Calprotectin [median (IQR)] (mg/kg)		82.3 [24.7–334]	129 [30.8–795]	0.438
C-reactive protein [median (IQR)] (mg/L)		2.1 [0.9–5.5]	1.4 [0.6–3.7]	0.514
Hemoglobin [median (IQR)] (g/dL)		12.8 [12.1–13.7]	14.5 [13.5–15.3]	<0.001
Ferritin [median (IQR)] (µg/L)		36 [23–62]	70 [32–119]	<0.001
Age [median (IQR)] (yrs)		38 [30–50]	40 [29–53]	0.843
Malnutrition Universal Screening Tool (MUST) [*n* (%)]	low risk	57 (48.7%)	71 (61.2%)	0.332
	medium risk	27 (23.1%)	22 (19%)	0.999
	high risk	33 (28.2%)	23 (19.8%)	0.808
Handgrip strength [median (IQR)]		28.9 [23.3–33.4]	46.8 [38.6–54.4]	<0.001
Diet quality Score [median (IQR)] (min = 0, max = 12)		6 [5–7]	6 [4–6]	<0.001
Diet diversity score [median (IQR)] (min = 0, max = 12)		5 [5,6]	5 [5–7]	0.027
Mediterranean diet score [median (IQR)] (min = 0, max = 9)		4 [3–5]	4 [3–5]	0.030
Screening Questionnaire of Highly Processed Food Consumption (sQ-HPF) [median (IQR)]		6 [5–8]	6 [5–9]	0.177
Estimated energy intake [median (IQR)] (kJ/d)		6433 [4770–8952]	7951 [5797–11,100]	0.004
Body mass index (BMI) [median (IQR)] (kg/m^2^)		23.8 [21.5–28]	24.4 [21.2–27.8]	0.895

Demographic data are expressed as total counts and percentages (*n* [%]) or as median values with the interquartile range (Md [IQR]). Statistical significance was ascertained through the implementation of either a Student’s *t*-test or Fisher’s exact test.

**Table 2:** Association between food-related quality of life and diet quality in men and women with IBD.

				FR-QoL-29																
				Low [<=73]						Medium [74–93]						High [94+]				
		Sex	*n*	Mean	SD	SEM	*p*	g	*n*	Mean	SD	SEM	*p*	g	*n*	Mean	SD	SEM	*p*	g
Diet Diversity Score [DGE]		women	45	5.3	1.8	0.3	0.548	−0.1	39	5.6	0.9	0.1	**0.044**	−0.5	33	5.6	1	0.2	0.204	−0.3
		men	34	5.5	1.6	0.3			39	6.1	1.2	0.2			43	6	1.4	0.2		
Diet Quality Score [DGE]		women	45	5.8	1.5	0.2	**0.019**	0.5	39	5.4	1.5	0.2	**0.003**	0.7	33	5.2	1.5	0.3	0.226	0.3
		men	34	4.9	1.8	0.3			39	4.5	1.3	0.2			43	4.8	1.5	0.2		
Mediterranean Diet Score [FFQ]		women	45	4	1.7	0.3	0.320	−0.2	39	3.9	1.7	0.3	0.839	0	33	3.6	1.5	0.3	**0.007**	−0.6
		men	34	4.4	1.6	0.3			39	4	1.6	0.3			43	4.6	1.5	0.2		
sQ-HPF (%)		women	45	36.1	7.2	1.1	0.216	−0.3	39	35.9	6.7	1.1	0.107	−0.4	33	35.9	7.3	1.3	0.669	0.1
		men	34	38.1	6.5	1.1			39	38.4	7.2	1.2			43	35.3	5.1	0.8		

The results of the Student’s *t*-test, which was conducted to analyze the disparities between men and women in the FR-QoL-29 percentiles (*Low*, *Medium*, and *High*), are reported as arithmetic mean, standard deviation (SD), standard error of the mean (SEM), level of significance (*p*), and the estimated effect size (g). The *p*-value of each *t*-test is printed in bold font when significant. DGE—German Nutrition Society; FFQ—food frequency questionnaire; sQ-HPF—Screening Questionnaire of Highly Processed Food Consumption.

**Table 3:** Association between food-related quality of life and food groups in men and women with IBD.

				FR-QoL-29																
				Low [<=73]						Medium [74–93]						High [94+]				
		Sex	*n*	Mean	SD	SEM	*p*	g	*n*	Mean	SD	SEM	*p*	g	*n*	Mean	SD	SEM	*p*	g
Fruits and vegetables (g/d)—FFQ *		women	45	346.8	337.4	50.3	0.169	0.3	39	287.7	235.6	37.7	**0.043**	0.5	33	335.8	301.1	52.4	0.400	0.2
		men	34	257.8	231	39.6			39	191.3	174.2	27.9			43	279.9	272.4	41.5		
Fruits and vegetables (EN%)—FFQ *		women	45	9.5	7.7	1.1	**0.036**	0.5	39	8.7	7.5	1.2	0.054	0.4	33	10.1	8.9	1.5	0.140	0.3
		men	34	6.2	5.3	0.9			39	5.9	5.3	0.8			43	7.6	5.4	0.8		
Nuts and seeds (g/d)—FFQ *		women	45	8.5	14	2.1	0.640	0.1	38	9	16.5	2.7	**0.043**	0.5	33	5.1	8.8	1.5	0.522	0.1
		men	34	7.2	10.7	1.8			39	3.2	5.2	0.8			43	3.9	6.9	1		
Nuts and seeds (EN%)—FFQ *		women	45	2.7	4.1	0.6	0.521	0.1	38	3	5.2	0.8	**0.033**	0.5	33	2	3.6	0.6	0.258	0.3
		men	34	2.1	3.7	0.6			39	1	1.5	0.2			43	1.2	1.8	0.3		
Cereal products (g/d)—FFQ *		women	45	158.6	117	17.4	**0.012**	−0.6	39	152.8	88.8	14.2	0.719	−0.1	33	159.7	101.4	17.6	0.106	−0.4
		men	34	257.3	195.8	33.6			39	160.5	99.3	15.9			43	197.5	98.8	15.1		
Cereal products (EN%)—FFQ *		women	45	18.1	9.3	1.4	0.089	−0.4	39	17.4	7.2	1.1	0.949	0	33	17.9	6.8	1.2	**0.045**	−0.5
		men	34	22.4	12.6	2.2			39	17.5	9.8	1.6			43	21.8	9.3	1.4		
Meat (g/d)—FFQ *		women	45	66.9	92	13.7	0.079	−0.4	39	72.3	100.6	16.1	0.069	−0.4	33	65.8	45	7.8	**0.037**	−0.5
		men	34	115.1	147.5	25.3			39	107.3	62.6	10			43	91.5	56.9	8.7		
Meat (EN%)—FFQ *		women	45	9.6	10.9	1.6	0.795	−0.1	39	9.4	8.9	1.4	**0.037**	−0.5	33	8.8	6.3	1.1	**0.033**	−0.5
		men	34	10.3	11	1.9			39	13.5	8.2	1.3			43	12.2	7.3	1.1		
Fish (g/d)—FFQ *		women	45	14.8	23.5	3.5	0.981	0	39	13.8	13.1	2.1	0.649	−0.1	33	10	8.2	1.4	**0.019**	−0.5
		men	34	14.9	17.8	3			39	15.6	21.9	3.5			43	17.6	18.3	2.8		
Fish (EN%)—FFQ *		women	45	1.7	2.3	0.3	0.475	0.2	39	1.6	1.5	0.2	0.707	0.1	33	1.2	1.2	0.2	0.120	−0.4
		men	34	1.3	2	0.3			39	1.5	1.5	0.2			43	1.8	1.8	0.3		
Spreadable fats (g/d)—FFQ *		women	44	3.7	4.5	0.7	0.075	−0.5	38	5.2	6.8	1.1	**0.039**	−0.5	33	5.5	9.8	1.7	0.515	−0.1
		men	33	9	16.1	2.8			39	9.7	11.5	1.8			43	6.7	6.5	1		
Spreadable fats (EN%)—FFQ *		women	44	1.7	1.9	0.3	0.116	−0.4	38	2.2	3.3	0.5	0.162	−0.3	33	1.6	1.9	0.3	0.068	−0.4
		men	33	3	5.2	0.9			39	3.4	4.2	0.7			43	2.6	2.5	0.4		
Eggs (g/d)—FFQ *		women	44	15.2	13.8	2.1	**0.010**	−0.7	39	24.4	28.6	4.6	0.560	−0.1	33	18.1	16.6	2.9	0.632	−0.1
		men	34	39.9	51.3	8.8			39	27.9	23.4	3.7			42	20	18.4	2.8		
Eggs (EN%)—FFQ *		women	44	1.4	1.4	0.2	0.055	−0.5	39	2.1	2.6	0.4	0.959	0	33	1.6	2	0.4	0.873	0
		men	34	3	4.5	0.8			39	2.2	1.8	0.3			42	1.6	1.6	0.2		
Dairy (g/d)—FFQ *		women	45	242.5	247.2	36.8	0.857	0	39	255.2	176.9	28.3	0.388	−0.2	33	252.5	217.6	37.9	0.752	−0.1
		men	34	253.2	274.4	47.1			39	313.3	377.6	60.5			43	274.8	354.8	54.1		
Dairy (EN%)—FFQ *		women	45	12	9.2	1.4	0.410	0.2	39	13.6	9.1	1.5	0.447	0.2	33	13	9	1.6	0.659	0.1
		men	34	10.5	7.1	1.2			39	12.1	8.3	1.3			43	12.1	7.8	1.2		
Sweet snacks (g/d)—FFQ		women	45	94.4	97.5	14.5	0.559	−0.1	39	101.2	138.3	22.1	0.500	0.2	33	127.4	102.6	17.9	0.120	0.4
		men	34	108.5	115.1	19.7			39	83.3	90.2	14.4			43	93.1	87.3	13.3		
Sweet snacks (EN%)—FFQ		women	45	20.2	14.8	2.2	0.128	0.3	39	19.8	15.8	2.5	0.057	0.4	33	23.5	14.5	2.5	0.011	0.6
		men	34	15.4	12.7	2.2			39	13.8	10.6	1.7			43	15.9	10.8	1.6		
Savory snacks (g/d)—FFQ		women	45	11.6	19.8	2.9	0.948	0	38	7.6	11.5	1.9	0.340	−0.2	33	9.5	18	3.1	0.814	−0.1
		men	34	11.9	16.8	2.9			39	10.8	17.1	2.7			43	10.4	14.2	2.2		
Savory snacks (EN%)—FFQ		women	45	2.6	3.9	0.6	0.747	0.1	38	2	2.4	0.4	0.654	−0.1	33	2	3	0.5	0.413	−0.2
		men	34	2.4	3.1	0.5			39	2.2	2.8	0.5			43	2.6	3.3	0.5		

The results of the Student’s *t*-test, which was conducted to analyze the disparities between men and women in the FR-QoL-29 percentiles (*Low*, *Medium*, *High*), are reported as arithmetic mean, standard deviation (SD), standard error of the mean (SEM), level of significance (*p*), and the estimated effect size (g). The *p*-value of each *t*-test is printed in bold font when significant. * Data were taken from FFQ, and the group definition was determined by DGE. EN%—energy percentage; DGE—German Nutrition Society; FFQ—food frequency questionnaire; sQ-HPF—Screening Questionnaire of Highly Processed Food Consumption.

**Table 4:** Association between food-related quality of life and macronutrients in men and women with IBD.

				FR-QoL-29																
				Low [<=73]						Medium [74–93]						High [94+]				
		Sex	*n*	Mean	SD	SEM	*p*	g	*n*	Mean	SD	SEM	*p*	g	*n*	Mean	SD	SEM	*p*	g
Estimated energy intake (kJ/d)—FFQ		women	45	7289	3963	590	**0.009**	−0.6	39	7285	3569	571	0.158	−0.3	33	7437	3683	641	0.491	−0.2
		men	34	10,531	6083	1043			39	8391	3270	523			43	7991	3272	499		
Carbohydrates (g/d)—FFQ		women	45	231	139.1	20.7	**0.018**	−0.6	39	208.3	105.4	16.9	0.116	−0.4	33	227.5	124.1	21.6	0.707	−0.1
		men	34	329.8	204.2	35			39	248.3	116.8	18.7			43	237.8	113.7	17.3		
Carbohydrates (EN%)—FFQ		women	45	52.9	10	1.5	0.915	0	39	49.2	9.4	1.5	0.772	−0.1	33	51.9	8.1	1.4	0.358	0.2
		men	34	52.7	9.4	1.6			39	49.8	8.8	1.4			43	50.2	7.7	1.2		
Fat (g/d)—FFQ		women	45	58.9	40.9	6.1	**0.012**	−0.6	39	66.5	42.7	6.8	0.547	−0.1	33	64.6	39.4	6.9	0.656	−0.1
		men	34	88.2	60.5	10.4			39	71.6	32.2	5.2			43	68.2	31.3	4.8		
Fat (EN%)—FFQ		women	45	30	8	1.2	0.593	−0.1	39	32.8	7.2	1.2	0.425	0.2	33	31.1	7.1	1.2	0.665	−0.1
		men	34	30.9	7.6	1.3			39	31.4	7.4	1.2			43	31.7	6	0.9		
Protein (g/d)—FFQ		women	45	62.2	30.4	4.5	**0.007**	−0.7	39	67.6	35.7	5.7	0.297	−0.2	33	62.7	24.3	4.2	0.107	−0.4
		men	34	90.3	51.6	8.8			39	75.3	27.8	4.5			43	73	29	4.4		
Protein (EN%)—FFQ		women	45	15.4	4.2	0.6	0.854	0	39	16.1	3.9	0.6	0.770	0.1	33	15.3	3.9	0.7	0.523	−0.2
		men	34	15.2	3.9	0.7			39	15.8	3.2	0.5			43	15.8	2.4	0.4		
Animal protein (g/d)—FFQ		women	45	34.3	20.8	3.1	**0.011**	−0.6	39	40	30.3	4.8	0.167	−0.3	33	35.6	15.7	2.7	0.083	−0.4
		men	34	53.5	38.1	6.5			39	48.6	23.5	3.8			43	43.5	21.9	3.3		
Animal protein (EN%)—FFQ		women	45	8.8	4.8	0.7	0.956	0	39	9.5	4.6	0.7	0.453	−0.2	33	9	4.1	0.7	0.532	−0.1
		men	34	8.9	4.3	0.7			39	10.2	3.4	0.5			43	9.5	3	0.5		
Fiber (g/d)—FFQ		women	45	18.6	12.5	1.9	0.272	−0.2	39	17.2	9.5	1.5	0.471	0.2	33	18.2	10.3	1.8	0.599	−0.1
		men	34	21.8	12.5	2.1			39	15.9	6.3	1			43	19.5	10.1	1.5		
Fiber (EN%)—FFQ		women	45	2	0.8	0.1	0.249	0.3	39	2	1	0.2	**0.025**	0.5	33	2.1	0.9	0.1	0.625	0.1
		men	34	1.8	0.8	0.1			39	1.6	0.6	0.1			43	2	0.7	0.1		
Estimated ethanol intake (g/d)—FFQ		women	45	11.9	26.7	4	0.630	−0.1	39	12.9	20.3	3.3	0.342	−0.2	33	6.5	10.7	1.9	**0.004**	−0.6
		men	34	15	29.8	5.1			39	18	26.5	4.2			43	60.9	117.6	17.9		
Estimated ethanol intake (EN%)—FFQ		women	45	6.2	18.7	2.8	0.998	0	39	5.2	7.5	1.2	0.385	−0.2	33	2.7	4.6	**0.005**	0.8	−0.6
		men	34	6.2	15.7	2.7			39	7	10.9	1.7			43	23.1	45.1		6.9	

The results of the Student’s *t*-test, which was conducted to analyze the disparities between men and women in the FR-QoL-29 percentiles (*Low*, *Medium*, and *High*), are reported as arithmetic mean, standard deviation (SD), standard error of the mean (SEM), level of significance (*p*), and the estimated effect size (g). The *p*-value of each *t*-test is printed in bold font when significant. EN%—energy percentage; FFQ—food frequency questionnaire.

**Table 5:** Logistic regression model (adjusted) on high IBD food-related quality of life in men.

		IBD Men—Outcome: High Food-Related Quality of Life		
		*n*	Odds Ratio [95% CI]	*p*
Entity	Crohn’s Disease	62	0.2 [0.08–0.74]	0.013
	Ulcerative Colitis (1)	47		
Disease Status	Remission (1)	59	0.1 [0.03–0.28]	<0.001
	Active Disease	50		

The findings of the logistic regression analysis, which has been adjusted, are expressed as the odds ratio (OR), the 95% confidence interval (CI), and the level of significance (*p*). The following adjustment factors were employed in the construction of the fully adjusted model: disease entity, age, animal/plant protein ratio, weight, handgrip strength, estimated GI, diet quality, diet diversity, MDS, disease status, and sQHPF. GI—glycemic index; MDS—Mediterranean diet score; sQHPF—Screening Questionnaire of Highly Processed Food Consumption.

**Table 6:** Logistic regression model (adjusted) on high IBD food-related quality of life in women.

		IBD Women—Outcome: High Food-Related Quality of Life		
		*n*	Odds Ratio [5% CI]	*p*
Disease Status	Remission (1)	57	0.1 [0.02–0.23]	<0.001
	Active Disease	51		
Weight (kg)		108	0.9 [0.91–0.99]	0.025
Age (years)		108	1.1 [1.01–1.10]	0.010

The findings of the logistic regression analysis, which has been adjusted, are expressed as the odds ratio (OR), the 95% confidence interval (CI), and the level of significance (*p*). The following adjustment factors were employed in the construction of the fully adjusted model: disease entity, age, animal/plant protein ratio, weight, handgrip strength, estimated GI, diet quality, diet diversity, MDS, disease status, and sQHPF. GI—glycemic index; MDS—Mediterranean Diet Score; sQHPF—Screening Questionnaire of Highly Processed Food Consumption.

## Data Availability

The original contributions presented in the study are included in the article; further inquiries can be directed to the corresponding author.
